# Soluble Guanylate Cyclase As the Key Enzyme in the Modulating Effect of NO on Metabotropic Glutamate Receptors

**Published:** 2018

**Authors:** I. V. Ryzhova, A. D. Nozdrachev, T. V. Tobias, E. A. Vershinina

**Affiliations:** Pavlov Institute of Physiology, Russian Academy of Sciences, Makarova Embankment 6, St. Petersburg, 199034, Russia

**Keywords:** nitric oxide, metabotropic glutamate receptors, vestibular apparatus, soluble guanylate cyclase, synaptic plasticity

## Abstract

The synaptic plasticity of the afferent synapse of the vestibular apparatus is defined by the dynamic interaction of ionotropic and metabotropic glutamate receptors and the modulators of synaptic transmission. It was shown that nitric oxide modulates iGluR responses. In this paper, the effect of NO on the function of the afferent synapse mGluR was investigated. Inhibitor of nitric oxide synthase lowered the level of background activity but increased the amplitude of the responses of groups I and II mGluR agonist ACPD. Donor NO SNAP increased the level of background activity. Short-term perfusion of the synaptic region with low concentrations of SNAP led to a decrease in the amplitude of the answers of mGluR agonists ACPD and DHPG. The inhibitory effect of the NO donor was eliminated under blockade of soluble guanylate cyclase with a specific inhibitor ODQ. A prolonged application of NO did not cause a statistically significant change in the amplitude of the ACPD response. However, SNAP at concentrations of 10 and 100 μM increased the amplitude of the mGluR agonist responses 30 and 15 minutes, respectively, after termination of the NO donor exposure. The obtained data show the multidirectional effect of NO on the function of mGluR and testify to the existence of a complex modulating mechanism of the afferent flow from vestibular organs to the central nervous system.

## INTRODUCTION

Hair cells, the secondary sensory receptors of the inner ear, contact the afferent fibers through an amino acid synapse. Glutamatergic synaptic plasticity is provided by the functional cross-talk between ionotropic and metabotropic glutamate receptors and the modulators of synaptic transmission released by hair cells, nerve terminals, or transported with the blood flow. Nitric oxide is of particular interest; it is a gaseous neuromodulator that functions as an intracellular and extrasynaptic signal messenger. *L*-arginine is the precursor of NO in humans and animals. NO-synthase and several cofactors catalyze the conversion of *L*-arginine in NO and *L-*citrulline. In the inner ear, nitric oxide can be synthesized under normal conditions by the neuronal and endothelial NO-synthases located in hair cells, afferent and efferent nerve fibers [1-3]. Pathological processes in the inner ear (labyrinthectomy, excitotoxicity, infections and exposure to ototoxic substances) are accompanied by the activation of inducible NOS, leading to prolonged synthesis of high NO concentrations [4-9].

The important role of NO in the functioning of the vestibular epithelium is confirmed by clinical and experimental physiological data. The baseline synthesis of NO in hair cells was detected by using a NO-selective electrode [10, 11]. NO content was increased in response to the action of acetylcholine, glutamate, antibiotics, liposaccharides and cytokines. The effects of NO donors and NOS inhibitors have been revealed in the vestibular epithelium of amphibians. It is shown that NO decreases the input resistance of vestibular afferent neuronal membranes, enhances the afferent resting discharge and increases the amplitude of the responses to adequate irritation and the magnitude of iGluR agonists’ responses. In contrast to the effects of NO donors, NOS inhibitors diminish the frequency of background activity in afferent fibers and the frequency of the excitatory postsynaptic potentials but increase the response amplitude of glutamate and its agonists, AMPA and NMDA [12-14]. There is a suggestion that NOS inhibitors act at the presynaptic level and suppress neurotransmitter release. We have shown the postsynaptic effect of NO donors and NOS inhibitors under conditions of blockade of the presynaptic membrane with a hypermagnesium-hypocalcium solution [13]. Data on NO involvement in the afferent discharge and adaptive changes in the vestibular analyzer have been published [3, 8, 9, 11, 12, 14].

According to some researchers, the mechanism of NO action on synaptic transmission, synaptic plasticity, and neurodegenerative processes is associated with the modulation of ion channel activity in the plasma membrane [15-18]. In the vestibular epithelium of rats, using the patch-clamp method of whole and perforated cells, it has been shown that NO inhibits Ca^2+^ currents of the semicircular canal crista hair cells by nitrosylation of Ca^2+^ channels and activation of the sGCcGMP signaling pathway [18]. It has been shown that NO weakens Ca^2+^-oscillations in frog saccular hair cells and increases the amplitude of the transients required to evoke these oscillations. This effect is associated with the inhibition of Ca^2+^-dependent K^+^- and Ca^2+^-channels by the decreasing of their opening probability. Inhibition of K+-channel transients by NO donors and by the membrane-permeable analogue of cGMP – 8-bromo-cGMP was observed in type I hair cells in rat semicircular canals [19, 20].

It is known that iGluR and mGluR are co-localized on the postsynaptic membrane. Taking into account the synthesis of NO during activation of NMDA receptors and rapid diffusion of NO along the concentration gradient, we hypothesized a possible effect of NO on the function of mGluR. Our pilot studies showed that the NO donor SNAP reduced the response amplitude of ACPD, a mGluR agonist, while the NOS inhibitor L-NAME had the opposite effect [21]. This work is devoted to a detailed study of the NO effect on the function of mGluR.

## EXPERIMENTAL

**Experimental procedure**

Normal and pathological synaptic processes are associated with different concentrations of NO and different times of its action. A physiologycally functioning synapse is exposed to the short-term effects of low NO concentrations, whereas under pathological conditions a synapse is subjected to the long-term action of high NO concentrations. Our choice of an experimental model was based on data showing that the NO content in the utricle increases 5 min after the application of aminoglycoside antibiotic gentamicin [22]. Hence, we investigated 1) the short-term (1 min) and long-term (9 min) effects of NO donor SNAP (1–100 μM) on the response amplitude of the mGluR agonists ACPD and DHPG and 2) proved the specificity of the NO effect on the function of mGluR via blockage of sGC, the receptor for NO, using a specific inhibitor, ODQ.

The frog vestibular system is a unique model that allows one to investigate the patterns of synaptic transmission in the bouton-like synaptic terminals between the hair cells and the afferent nerve fibers. The cartilage capsule of the labyrinth was excised and placed for further dissection in a chamber with a solution of the following composition (in mM): NaCl 117; KCl 2.5; NaHCO_3_ 1.2; NaH2PO3 • 2H_2_O 0.17; CaCl_2_ 1.8; glucose 2.5. The tested substances were dissolved in a normal solution at the required concentrations (pH 7.4). The solutions were applied through external perfusion. The following substances manufactured by Sigma-Aldrich were used in the experiments: NO-synthase inhibitor L-NAME; NO donor SNAP; mGluR agonists: group I and II – ACPD, and selective group I (mGlu1, mGluR5) - DHPG; and selective sGC inhibitor ODQ.

The multiple impulse activity of the afferent fibers contacting the hair cells of the semicircular canal was recorded with a suction glass electrode. The impulse activity was applied to an amplifier A-M Systems Inc 3000 and converted into standard rectangular pulses of 2 ms duration, which were recorded on a computer online throughout the experiment using the original program. The responses of the mGluR agonists ACPD and DHPG were estimated as the ratio of the difference between the maximum and minimum of the response to the resting discharge rate (frequency, (max – min) of resting discharge, %). We compared the change in the response amplitude of the mGluR agonists ACPD and DHPG before, combined and after application of the NOS inhibitor and NO donor SNAP.

The data are presented as mean values and standard errors of the mean (M ± SEM). Data analysis was performed using one-way ANOVA for dependent variables (Repeated measurements) followed by multiple pair comparisons (Post hoc test). The factor is the stage of the process (control answer/application/recovery). In the absence of sufficient material in the recovery stage, only two stages were compared using the *t*-test. All calculations were duplicated with the Wilcoxon rank test for dependent variables. A statistical decision was accepted at a 5% level of significance. Estimations were performed using the SPSS Inc. software complex. Illustrations were prepared using the MS-Excel software package.

## RESULTS

**The influence of NO donor SNAP on the background activity in afferent fibers**

**Fig. 1:**
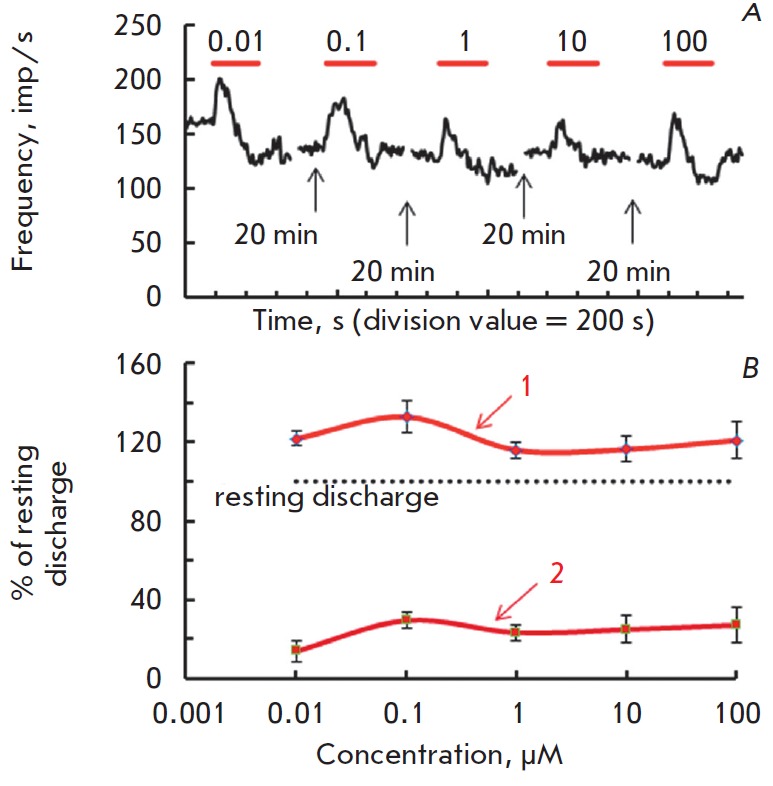
Effects of long-term application of different concentrations of NO donor SNAP on the background activity in posterior semicircular canal nerve fibers of a frog. *A *– original recording of firing activity in the same experiment. Ordinate: spike frequency, imp/s; abscissa: time, s. The horizontal lines above the recording indicate the duration of drug applications. *B *– dose-response curves of the positive (1) and the negative (2) response waves for NO donor SNAP. *N*=5–6

Perfusion of the synaptic area with a SNAP solution (0.01–100 μm) caused positive-negative changes in the frequency of the resting activity. The effect of the NO donor SNAP was characterized by a lack of dose-dependence. The dynamics of responses varied widely from one experiment to another, which was reflected in a different ratio of amplitude and duration of positive and negative response waves. Prolonged exposure to high SNAP concentrations (100 μM) slightly increased firing activity, followed by a decrease in the frequency of afferent discharges. Reduced level of firing activity was not restored to its initial level after 30-min exposure in a normal solution (*Fig. 1*).

**The short-term effect of the NO donor on the function of metabotropic glutamate receptors**

The effect of NO on the response amplitude of groups I and II mGluR agonist ACPD and group I mGluR agonist DHPG were investigated in our work, because only groups I and II mGluR were revealed in the vestibular epithelium [23, 24].

**Fig. 2:**
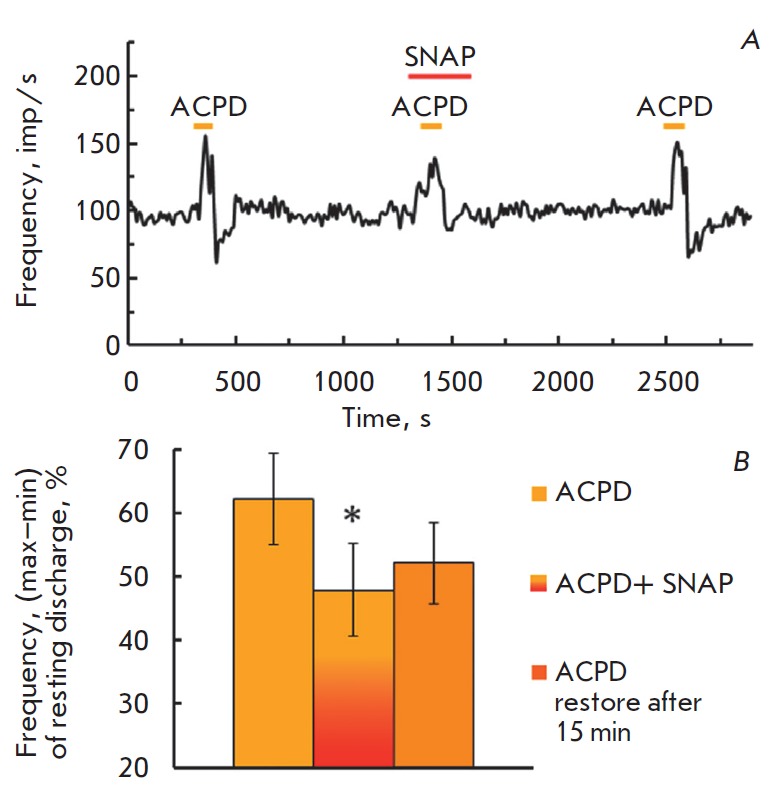
Modification of the ACPD-evoked response before, combined and a short-time impact of NO donor SNAP (ACPD 100 μM, SNAP 1 μM). *A – *time course of firing activity in a typical experiment. The designations are the same as in *Fig 1A*. *B – *diagram of the decrease of the ACPD-evoked response combined short-term SNAP application. Abscissa: left to right – ACPD-control; SNAP+ACPD; ACPD recovery after 15 min in a normal solution. Ordinate: value of the response to ACPD, %, mean ±SEM. (ANOVA F(2.18) = 3.9, *p *= 0.039, Post hoc test *p *= 0.03)

ACPD, the agonist mGluR, increased spike frequency. To discriminate the changes in the excitatory wave of mGluR agonist responses from the excitatory effect of NO donor SNAP on the resting activity, ACPD was applied when a significant effect of the NO donor occurred. The amplitude of the ACPD-evoked response during SNAP application was lower compared to the control answer before SNAP application. The observed changes were reversible after 15-min washing in a normal solution (*Fig. 2*).

**Fig. 3:**
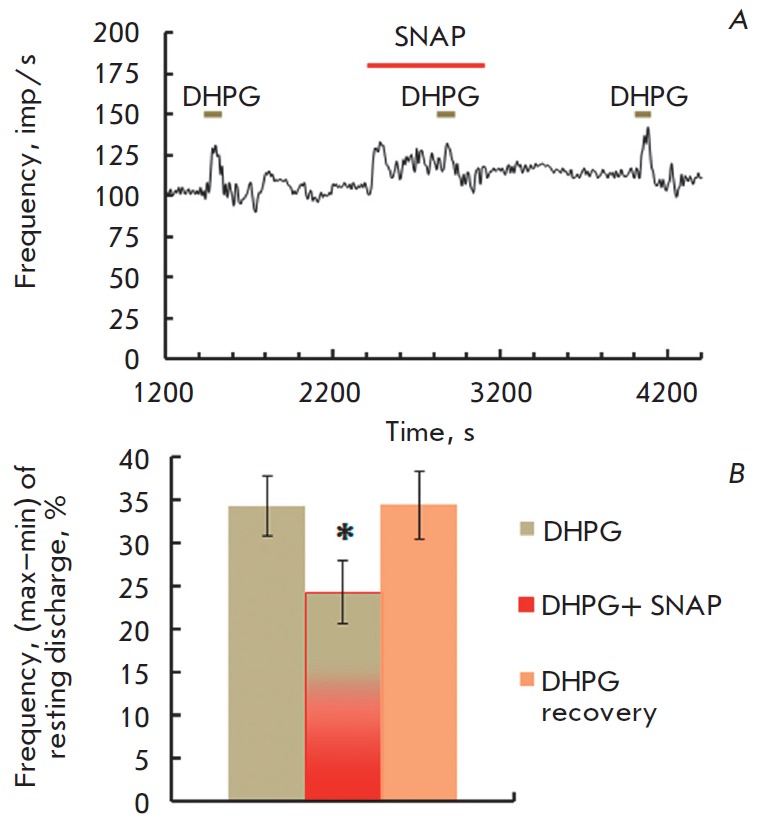
Effects of NO donor SNAP (1 μM) on the amplitude of mGluR I agonist DHPG (200 μM). *A* – typical recording of the experiment. The designations are the same as in *Fig. 1 A,B –* bar graph of reversible suppression of the DHPG-evoked response before, during and after SNAP application. Abscissa: left to right: DHPG – control; SNAP+DHPG; DHPG recovery in 15 min in a normal solution. Ordinate: mean ±SEM for amplitude of DHPG-induced responses (%). (Wilcoxon *p *= 0.025)

NO donor SNAP at low concentrations had a similar effect on the responses of the DHPG – group I mGluR specific agonist. DHPG application (200 μM) was accompanied by an increased spike frequency in afferent fibers. NO donor SNAP (1 μM) significantly reduced the amplitude of the group I mGluR agonist DHPG response (*Fig. 3*). Thus, the short-term impact of NO suppressed the function of groups I and II mGluR. The inhibitory effect was completely reversible.

**The long-term effect of NO on mGluR function**

Prolonged influence of NO to mGluR function is of particular interest due to the long-term effect of a high concentration of NO during pathological processes in the inner ear. In our experiments, the mGluR agonist ACPD (100 μM) was initially applied (control). After a 15-minute washing of the vestibular with a normal solution, a 5-minute perfusion of the NO donor SNAP (0.1–100 μM) was performed, against which the mGluR agonist ACPD was re-applied. Recovery of ACPD responses in a normal solution was monitored 15 and 30 minutes after cessation of the combined effect of SNAP and ACPD.

The long-term perfusion of the synaptic area with solutions of low SNAP concentration (0.1–1 μM) did not affect either the response value after 5-min perfusion or recovery of the response after 15 and 30 min. SNAP at a concentration of 10 μM did not change either the response of the mGluR agonist after a 5-min perfusion or the response amplitude of the mGluR agonist after 15-min washing in a normal solution. However, the amplitude of the ACPD-evoked response increased significantly 30 min after the end of SNAP (10 μM) action and amounted to 169.9% compared to the background activity. Tendencies to differences were observed between responses to combined ACPD and SNAP exposure and its recovery after 15 min (Post hoc test = 0.082) and 30 min (Post hoc test = 0.059).

**Fig. 4:**
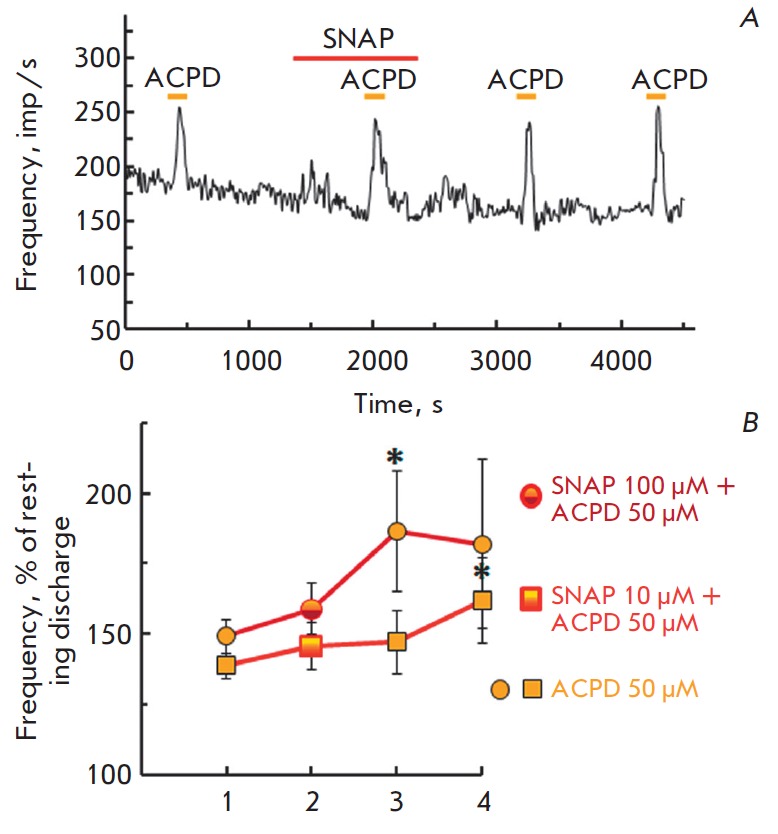
Effects of a prolonged application of NO donor SNAP on the amplitude of group I and II mGluR agonist ACPD (50 μM). *A – *original recording (SNAP 10 μM). The designations are the same as in *Fig. 1 A,B –* the increase in the amplitude of ACPD-evoked responses after prolonged exposure of different concentrations of NO donor SNAP in the vestibular. Abscissa: left to right: 1 – control; 2 – over 5-min application of SNAP; 3 – recovery 15 min after the end of drug application in normal solution; 4 – recovery in 30 min in a normal solution. Ordinate: mean ±SEM for the amplitude of the ACPD-induced response (%). SNAP 10 μM (ANOVA F(3.18) = 4.4, *p *= 0.017, Post hoc test *p *= 0.047) and SNAP 100 μM (ANOVA F(2.16) = 4.58, *p *= 0.027, Post hoc test *p *= 0.027)

A 5-min SNAP application (100 μM) did not change the amplitude of ACPD-evoked responces. But the ACPD induced response increased significantly in 15 min washing in a normal solution. This increase in the response amplitude remained at that level also 30 min after NO exposure, although it was statistically insignificant (*Fig. 4*).

Thus, the effect of NO on the mGluR function depended on the concentration and duration of the exposure. A short-term exposure to low NO concentrations inhibited the mGluR function. The long-term impact of high concentrations of the NO donor enhanced mGluR responses.

**The effect of a specific sGC inhibitor on the depressive effect of NO donor SNAP**

**Fig. 5:**
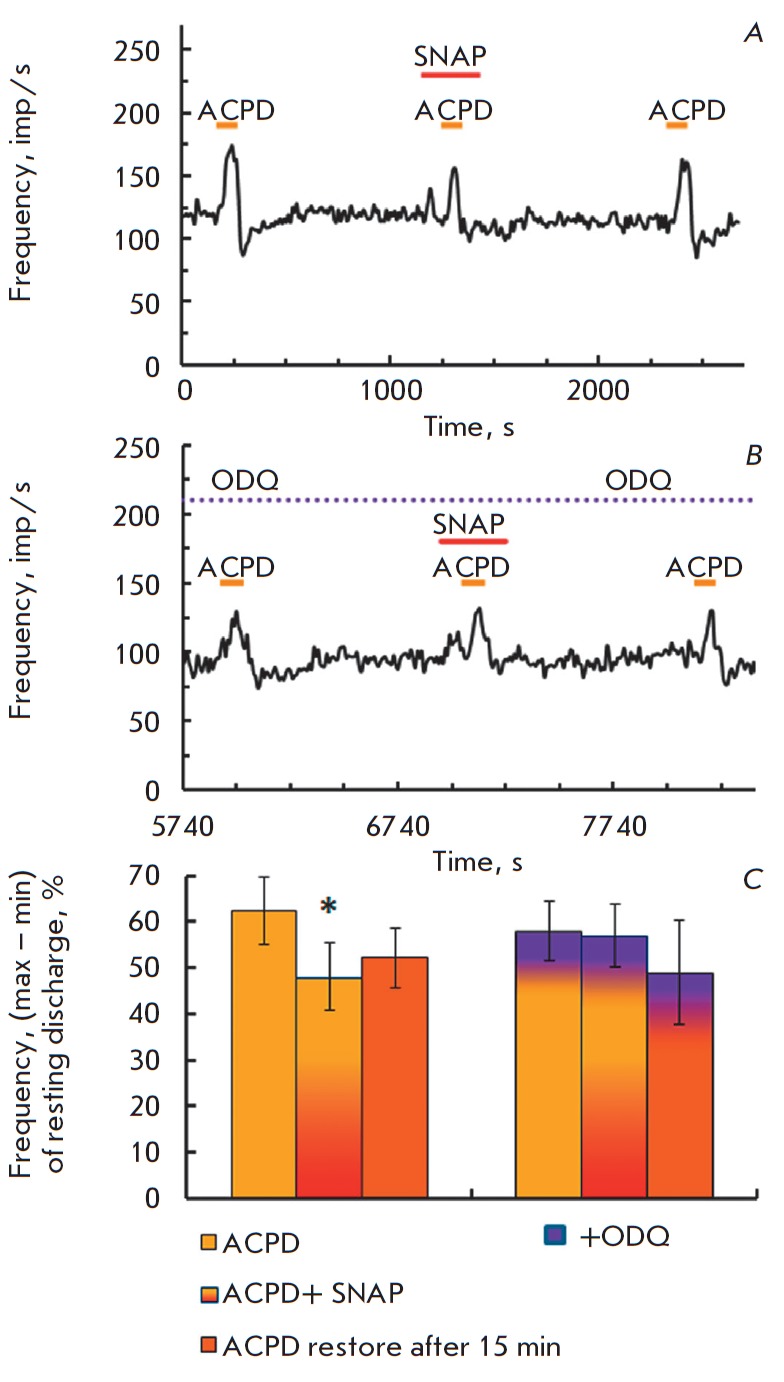
Effects of the specific inhibitor of sGC ODQ on SNAP (1 μM) suppression of the ACPD-induced response. *A, B – *multiunit recording of firing activity in the posterior semicircular canal nerve before (*A*) and after (*B*) a 20-min perfusion on vestibular synapse by 15 μM ODQ. The designations are the same as in Fig 1*A. C – *lack of suppression of ACPD-evoked responses after inhibition of sGC by ODQ. Abscissa: left to right: control; ACPD against SNAP; recovery 15 min after the end of drugs application in a normal solution; the same steps against ODQ application. Ordinate: mean ±SEM for the amplitude of the ACPD-induced response (%)

The following protocol was used to investigate the involvement of sGC in the modulation of the mGluR function. Step 1 (*Fig. 5A*): application of mGluR agonist ACPD (control 1), washing of the vestibular apparatus in a normal solution, perfusion of the synaptic area with a solution of SNAP (1 μM) plus ACPD (100 μM) (inhibitory effect, application 1), recovery of ACPD-evoked response after 15 min washing in a normal solution. Step 2 (*Fig. 5B*): 20-min incubation of vestibular in a solution of the specific sGC inhibitor ODQ (15 μM), then repeat stage 1 against ODQ perfusion [application of ACPD solution 100 μM (control 2), SNAP (1 μM) and ACPD solution (100 μM) (application 2), recording of the ACPD response after 15 min of washing with ODQ (recovery 2)]. Stage 3: perfusion of the vestibular apparatus for 1 h in a normal solution and testing of the preservation of the ODQ influence on the inhibitory effect of SNAP to the ACPD-induced response (step 1 was repeated: control 3, application 3, recovery 3). To investigate the ODQ influence on the inhibitory effect of SNAP to mGluR agonist, the differences in the amplitudes of ACPD-evoked responses were estimated between control 1 and application 1 between control 2 and application 2. Preservation of the ODQ effect was expressed as the difference between control 3 and application 3 of stage 3. ANOVA and pairwise multiple comparisons revealed a significant decrease in the amplitude of mGluR agonist ACPD under the action of the NO donor SNAP (ANOVA F(2.18) = 3.9, *p *= 0.039, Post hoc test – *p *= 0.03) that was abolished after blockade of sGC with the specific inhibitor ODQ (ANOVA F(2.20) = 0.408, *p *= 0.67). The effect of ODQ persisted for 1 hour *(Fig. 5)*.

## DISCUSSION

The functional role of mGluR in various CNS structures was studied in detail. mGluRs play a key role in CNS ontogenesis [25] and participate in the long-term potentiation, depression, learning, and formation of long-term memory [26-28]. mGluRs were revealed on glial cell membranes and on the pre- and postsynaptic membranes in the cortex, striatum, hippocampus, and cerebellum [26, 29-31]. To date, eight mGluR subtypes have been cloned. They are subdivided into three groups according to structure, pharmacological characteristics, and the second messengers involved [29, 32-34]. In all cases, the activation of mGluR is associated with Ca^2+^ release from intracellular stores and prolongation of the excitation wave triggered by iGluR.

There are two types of Ca^2+^-channels with different functional and pharmacological characteristics on endoplasmic reticulum membranes. Ryanodine receptors (RyR) functionally interact with the potential-dependent Ca^2+^-channels of the plasma membrane and are activated by low concentrations of ryanodine, ATP, heparin, and micromolar concentrations of Ca^2+^ cations. The function of the Ca^2+^-channels of the ryanodine receptor can be inhibited by millimolar concentrations of Ca^2+^ and ryanodine, be modulated by NO, oxidants, protein kinases, and intracellular proteins [35, 36]. The inositol trisphosphate receptors (IP3R) located on endoplasmic reticulum membranes are activated by IP3 but are inhibited by calmodulin and the NO/cGMP-activated kinase I [37].

Information on the mGluR functions and the ways they are modulated in the acousticolateral system is scarce. Only groups I and II mGluR located on the pre-and postsynaptic membranes have been found in the vestibular epithelium of amphibians. The function of the mGluR2 and mGluR3 subtypes has not been studied yet [23, 24]. Evidence of a direct involvement of mGluR I in afferent synaptic transmission in the vestibular apparatus [23] and in the cochlea [38, 39] was obtained using methods of electrophysiology, immunohistochemistry, and molecular genetics.

The participation of group I mGluR in the functioning of glutamatergic synapses was demonstrated in frog semicircular canals [23, 40]. In those studies, the mGluR I–II agonist ACPD and mGluR I specific agonist DHPG produced an increase in the afferent firing rates of the ampullar nerve. It was proved that activation of presynaptic mGluR facilitates glutamatergic transmission due to intracellular Ca^2+^ release from IP3-sensitive and ryanodine/caffeine-sensitive intracellular Ca^2+^-stores.

The functional relationship between group I mGluR and IP3R of the endoplasmic reticulum was revealed in the frog vestibular apparatus by immunocytochemistry and electrophysiology. The participation of IP3 in the modulation of the resting activity and mechanically evoked responses has also been proved [41]. The data observed show that the amphibian vestibular apparatus contains a heterogeneous population of mGluR the activation of which is associated with the formation of IP3, the activation of IP3 and ryanodine receptors, and Ca^2+^ release from endoplasmic reticulum cisterns. It is important to note that the ACPD-evoked response was caused by the activity of mGluR but was not due to the activity of ionotropic receptors, since specific iGluR antagonists did not change the amplitude of the ACPD response [23].

The afferent fibers of the vestibular epithelium produce a background activity, which reflects tonic release of glutamate from hair cells [42]. The sensitivity of the vestibular apparatus to adequate stimuli and informativity of the signal perceived by the CNS are mediated by the ratio between resting and evoked activity. According to current concepts, activation of mGluR in the vestibular epithelium of semicircular canals produces a positive feedback reinforcing the contrast between background and induced activity [23]. This hypothesis is confirmed by our data showing that application of the mGluR agonist ACPD increases the response amplitude of iGluR agonists [24].

The results of our pilot experiments and the presented data indicate that fluctuation of NO content can modulate the function of mGluR. In our experiments, NOS inhibitor L-NAME reduced afferent resting firing depending on its concentration but increased the response amplitude of ACPD [21]. These results confirm the hypothesis about the various mechanisms of background and evoked vestibular afferent firing [42]. Our data about the effect of NOS inhibitors on resting afferent nerve firing [13] are in line with the results received in various models of the acousticolateral system: L-NAME inhibits the basal spike discharge in lateral line cells [43]. The NOS inhibitors L-NOARG and L-NAME elicited a decrease in the basal discharge and diminished the EPSP rates of the axolotl vestibular system [1, 12]. Using a specific fluorescent probe, the synthesis of NO was shown to be decreased in the presence of L-NAME in the vestibular epithelium of the frog saccule [11]. This data allows us to conclude that a small amount of NO is synthesized in the vestibular epithelium under normal physiological conditions. The decrease in resting activity and increase in the ACPD response amplitude under NOS blockade with the inhibitor L-NAME are specific.

The inhibitory effect of low NO donor concentrations on the mGluR agonists responses was studied with simultaneous and consecutive application of NO and mGluR agonists. This allowed us to separate the stimulating effect of mGluR agonists from the exitation effect of the NO donor on basal activity and to compare the effect of NO donor SNAP on iGluR and mGluR. Our data showed that simultaneous short-term application of the NO donor increased the response amplitude of the iGluR agonists AMPA and NMDA [14] but reduced the response magnitude of the mGluR agonist ACPD. The inhibitory effect of SNAP on the ACPD-evoked response was dose-independent.

It was shown that NO can modify nerve cell excitation through two basic mechanisms: direct interaction with the ion channel protein (nitrosylation reaction) and by activating the NO/sGC/cGMP/PKG signaling cascade [15, 44]. Both pathways have been found in the hair cells of the inner ear [18]. To study the participation of soluble guanylate cyclase, and the possible involvement of the NO/sGC/cGMP signaling pathway in the inhibitory effect of NO on mGluR, we used the specific sGC blocker ODQ, since sGC is the specific cytosolic receptor for NO [45].

In our experiments, the specific blocker of sGC ODQ eliminated the inhibitory effect of NO donor SNAP, suggesting an involvement of the NO-sGC-cGMP signaling cascade in mGluR modulation. We failed to find direct data on the impact of NO on the mGluR function. However, it was found that NO can inhibit the G-protein-mediated signaling pathway. The possibility of IP3 receptor phosphorylation and a decrease in the intracellular Ca^2+^ concentration by the activation of PKG was shown in Chinese hamster ovary cells. In those experiments, increase in the intracellular Ca^2+^ concentration caused by the activation of the thrombin/G-protein/phospholipase C signaling cascade was fully prevented by 8-bromo-cGMP, which indicates a specific effect of cGMP-stimulated kinase [46].

Similar data were obtained on cell cultures transfected with IP3R and incubated in the presence of the cGKI-specific cGMP analogue 8-pCPT-cGMP. Pre-incubation of cells expressing IP3R, the IRAG complex and cGKIβ protein kinase with the specific analog 8-pCPT-cGMP, reduced bradykinin-stimulated release of Ca^2+^ from intracellular stores. The observed effect was linked to the phosphorylation of the IRAG protein complex and reduced IP3 synthesis [37].

Different experimental models revealed cGMP-dependent and cGMP-independent routes of NO impact on sarcoplasmic reticulum receptors. It is assumed that NO can modify ryanodine and IP3 receptor functions. These two mechanisms differ in the activation kinetics and NO concentrations required for modulation. The NO-activated enzymatic pathway leading to PKG activation and inhibition of IP3 synthesis (or phosphorylation of IP3 receptors) is triggered by low NO concentrations within seconds. Nitrosylation of RyR is initiated by a higher concentration of NO, develops within several minutes, and is associated with a higher activity of the Ca^2+^-channel connected with an increase in the probability of RyRs opening [47, 48]. Thus, efflux of Ca^2+^ ions from intracellular stores can be modulated in time through various mechanisms. The long-term exposure to high NO concentrations can cause nitrosylating stress, leading to pathology [47, 49].

Our data support the hypothesis that NO can modify the mGluR function withal the modulating effect depending on the concentration and time of NO action. Short-term exposure to low NO concentrations suppresses mGluR agonist responses and, consequently, decreases Ca^2+^ efflux from intracellular stores. In our opinion, inhibition of the mGluR function with low NO concentrations can be proposed as one of the mechanisms of glutamatergic synaptic plasticity aimed at decreasing positive feedback [24]. Prolonged activation of the NO signaling cascade with high concentrations of a NO donor causes a low but statistically significant increase in the amplitude of the mGluR agonist response that may be hypothetically associated with the activation of ryanodine receptors. These assumptions require more experimental confirmation.

Thus, in this paper we showed the following:

NO affects the function of agonists of different mGluR groups (ACPD and DHPG).

Blocking a specific NO receptor eliminates the inhibitory effect of NO on the mGluR function, which suggests specificity of the NO influence. (We plan to search for evidence of the involvement of different links in the NO pathway in the modulation of the mGluR function in further experiments.)

The direction of the NO effect on the mGluR function is dynamic and depends on the concentration and time of influence.

## Abbreviations

iGluR: ionotropic glutamate receptors
mGluR: metabotropic glutamate receptors
NO: nitric oxide
NOS: nitric oxide synthase
L-NAME: N-nitro-L-arginine methyl ester hydrochloride
sGC: soluble guanylate cyclase
cGMP: cyclic guanosine monophosphate
SNAP: S-Nitroso-N-acetyl-DL-penicillamine,
IP3R: inositol trisphosphate receptors
RyR: ryanodine receptors
PKG: protein kinase G
ODQ: 1H-[1,2,4] Oxadiazolo[4,3-a]quinoxalin-1-one
ACPD: 1S,3R)-1-aminocyclopentane-trans-1,3-dicarboxylic acid
DHPG: (S)-3,5-dihydroxyphenylglycine
AMPA: α-amino-3-hydroxy-5-methyl-4- isoxazolepropionic acid
NMDA: N-methyl-D- aspartate
CNS: central nervous system
EPSP: excitatory postsynaptic potential
